# Analysis of Cross-Border Movement of Cattle in Yunnan Province, China: Insights Into Transboundary Animal Diseases Control

**DOI:** 10.1155/tbed/5561414

**Published:** 2025-09-11

**Authors:** Dai Meixia, Yi Ying, Tang Hao, Liu Hanze, Yang Honglin, Li Chao, Shen Chaojian, Zhang Yi

**Affiliations:** ^1^Institute of Zoonosis, College of Public Health, Zunyi Medical University, Zunyi, Guizhou, China; ^2^Key Laboratory of Maternal and Child Health and Exposure Science of Guizhou Higher Education Institutes, Zunyi, Guizhou, China; ^3^China Animal Health and Epidemiology Center, Qingdao, Shandong, China; ^4^Qingdao Municipal Center for Disease Control and Prevention, Qingdao, Shandong, China; ^5^Biosecurity and One Health Research Centre, Harry Butler Institute, Murdoch University, Perth, Western Australia, Australia; ^6^School of Veterinary Medicine, Murdoch University, Perth, Western Australia, Australia

**Keywords:** cattle, China, cross-border animal movement, risk management, Southeast Asia, transboundary animal diseases

## Abstract

Owing to the unique geographical location of Yunnan province in China, cross-border animal movement has historically been frequent. To get the routing and amount of cross-border animal movement and assess the impact of border control infrastructures on animal movement and potentially disease transmission, a cross-sectional study was conducted in the border areas of Yunnan province from August to October 2023. Epidemiological survey data showed that the smuggled cattle came from Myanmar, Laos, Thailand, India, Bengal, Sri Lanka, and other countries, and then the cattle were cross-border transported through different county, and by several different pathways. Due to the isolating devices on the border and narrowing price gap, the volume of cross-border animal movements sharply decreased compared to that before the COVID-19 pandemic. Most of the smuggled cattle were sold to the southern provinces of China, a proportion of them were short-term fattened locally. In the process of cross-border and domestic transport, the practice of no disinfection of the transport vehicle and ship and mixing of cattle from different origins were high-risk practices for disease transmission. In the livestock market, no cleaning and disinfection, sharing forage and cattle sheds, high density of people, vehicle, and cattle may accelerate disease transmission. This survey and analysis may serve as a foundation for risk control and intervention strategies of transboundary animal disease.

## 1. Introduction

Transboundary animal diseases (TADs) are a category of animal diseases that can disseminate to other countries or regions through the trade of animals and products, nomadic animals in transit, human interactions, and so on [1]. TADs are defined by the Food and Agriculture Organization (FAO) of the United Nations as highly contagious and transmissible epidemic diseases of livestock which have the capability for rapid spread to new areas and regions, regardless of national borders, and have serious socioeconomic and public health consequences [2]. These diseases can have profound consequences on the safety of animal husbandry, biosecurity, and public health in the recipient countries or regions [1]. As globalization continues to progress, the formal and informal movement of animals and their products is escalating, thereby heightening the risk of animal populations being exposed to TADs pathogens [3].

Examples of TADs include foot-and-mouth disease (FMD), lumpy skin disease (LSD), and so on. These diseases have the potential to rapidly spread across international borders, causing severe and sometimes catastrophic economic losses in nonendemic countries [4, 5]. Illegal imports are often proven or suspected to be the triggering factor for initial outbreaks of TADs in various countries [6]. Although formal trade activities carry some inherent risk of disease transmission, strict quarantine can greatly reduce and eliminate this risk. However, the risk associated with informal or illegal movements of animals and animal products from infected countries is significantly higher, and the risk is often difficult to estimate and control [3, 7]. FMD stands out as one of the most significant infectious livestock diseases, endemic in the majority of developing countries and exerting a notable impact on the global economy. Both legal and illegal trade in livestock and their products have been implicated in the spread of FMD between nations. In the pastoral environments of Africa, nomadic herders share grazing lands, watering points, and illicit markets between bordering countries or evade diseases or interethnic conflicts through cross-border movements [8], which are significant factors contributing to the transboundary spread of FMD in the African region. The outbreak of FMD in Taiwan is believed to have been most likely caused by the smuggling of pork or live animals from mainland China by fishing boats in 1997 [3, 9]. In a similar way, China reported five FMD outbreaks in 2019 alone, largely attributed to the smuggling of live cattle and beef [10–12]. LSD is an emerging and highly contagious transboundary viral disease of livestock that is caused by the lumpy skin disease virus (LSDV). Initially reported in Zambia in 1929, LSDV was initially confined to African countries. However, a series of cluster outbreaks have occurred across the Asian subcontinent [13, 14], with thousands of LSD outbreaks reported in Southeast Asian countries since 2019 [15–17]. Epidemiological studies indicate that the cross-border spread of LSD to nonendemic countries is attributed to various risk factors, including both legal and illegal livestock transport and trade, the movement of insect vectors and hosts across borders, the slowdown of vaccination campaigns, and diminished global surveillance efforts [18]. Notably, LSD was first reported in China in August 2019 [15, 19], followingly widely spreading and leading to the mass deaths of cattle [13], and investigations have indicated that outbreaks in several provinces and autonomous prefectures are likely linked to the introduction of live cattle trade and the frequent smuggling of frozen beef in recent years [20]. Given its potentially high transboundary infectivity and negative economic impact, the World Organization for Animal Health (WOAH) has identified LSD as an important transboundary reportable disease [13, 21]. Avoiding or minimizing the cross-border transmission of zoonotic diseases has become a critical concern [22].

China stands as a prominent producer and consumer of beef on the global stage, consistently holding its rank as the world's foremost importer and the second largest consumer of beef for numerous years. In terms of overall consumption, China's beef intake constituted 16.1% of the global aggregate in 2020, trailing only behind the United States. Given its vast population, China exhibits substantial total beef consumption, thereby emerging as a significant market for global beef consumption, characterized by a high demand for beef. This situation may render the smuggling of live cattle and beef in China economically viable, particularly when the prices of smuggled cattle are lower than those in the domestic market [23, 24].

Yunnan's strategic positioning as a pivotal trade corridor between Southeast Asia and China, in conjunction with its extensive border shared with Myanmar, Laos, and Vietnam, promotes a variety of cross-border animal movements and transactions. This geographical configuration may facilitate the spread of TADs and the emergence of infectious diseases [25]. Moreover, located in southwest China, Yunnan province stands out as a hotspot for insect-borne diseases due to its warm and rainy summers, ideal conditions for the proliferation of biting flies like mosquitoes and midges [26–28]. These insects serve as vectors for numerous TADs, facilitating their spread across borders. Collectively, these factors underscore Yunnan province's heightened vulnerability to TADs, necessitating a thorough analysis of cross-border animal movements in its border areas. In response to the COVID-19 pandemic, extensive physical barriers, such as fencing and blockades, were constructed along the border to prevent unauthorized movement of people and vehicles. However, the potential impact of these border control measures on cattle movements, particularly regarding the unofficial movements, remains insufficiently understood. To date, few studies have addressed how such interventions have influenced livestock flows across the border. This study aims to investigate changes in cross-border cattle movement in Yunnan's border areas before and after the onset of the COVID-19 pandemic. The findings would provide crucial insights into the transmission pathways and risk management of TADs in these border areas.

## 2. Materials and Methods

### 2.1. General Overview

From August to October 2023, an epidemiological investigation was conducted by the China Animal Health and Epidemiology Center, in collaboration with Zunyi Medical University and the Yunnan Center for Animal Disease Control and Prevention. The objective was to investigate the cross-border movements of cattle and associated activities contributing to TADs transmission in the border regions of Yunnan Province.

The study first identified key areas with high incidences of cross-border livestock movement. Data collection encompassed both the periods before and after the COVID-19 pandemic in these areas. Subsequently, the movement of cattle was investigated, focusing on the changes of the volumes, as well as the identification of cross-border illegal movement pathways. Ultimately, the study identified critical control points and practices present in the pathways that could affect the risks of TADs transmission.

### 2.2. Study Areas

**Figure 1:**
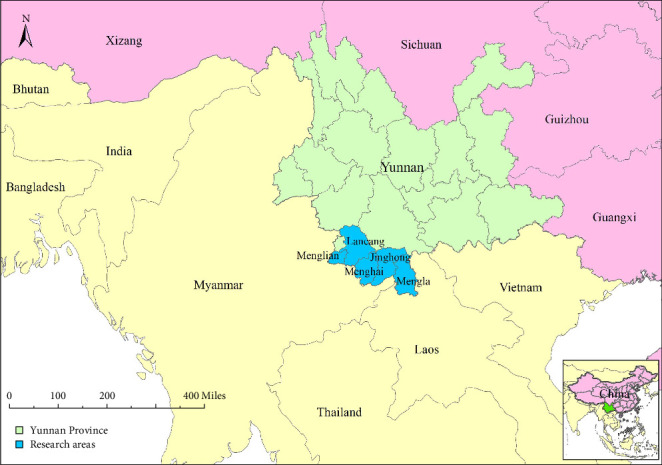
Geographical location of research areas (blue) of Yunnan Province (green). The research areas specifically encompass Mengla County, Menglong Town, Jinghong City, and Menghai County within the Xishuangbanna Dai Autonomous Prefecture, as well as Lancang County and Menglian County in Pu'er City.

This study was conducted in five counties located in border areas: Mengla County, Menglong Town, and Menghai County in Xishuangbanna Dai Autonomous Prefecture; Lancang County, and Menglian County in Pu'er City. After consultation with the Yunnan Centre of Animal Disease Prevention and Control (CADC), these areas were purposively selected. These counties were chosen due to their proximity to international borders and the high frequency of cattle smuggling activities. According to local veterinary authority, more than half of the foreign cattle in Yunnan were cross-border transported through their surveyed counties, making them highly representative and significant [12]. The specific locations of these study areas are illustrated in Figure 1. The picture shows that Mengla County mainly borders Laos, while Menglong Town in Jinghong City, Menghai County, Lancang County, and Menglian County mainly border Myanmar.

### 2.3. Data Collection

**Figure 2:**
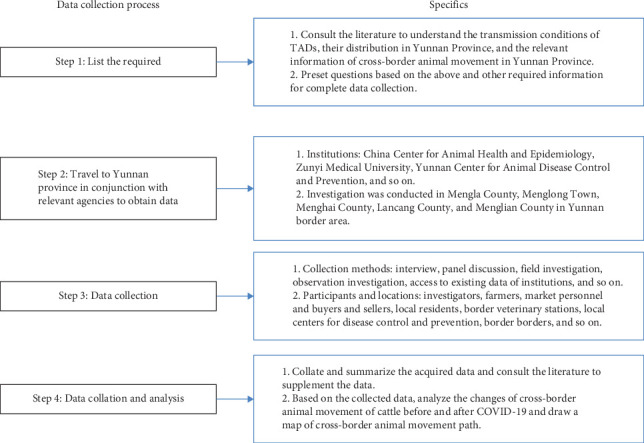
Data sources. The data collection processes used in the study and the specific content of each process.

Focus group discussions (FGDs), key informant interviews (KIIs), and on-site observations were conducted in the study areas to collect data. FGDs focused on mapping the cross-border paths of the cattle and the movement and trade of the cattle in China, including identifying stakeholders and premises involved and describing their profile, flows, and interactions. While KIIs collected qualitative and quantitative data on stakeholders' practices and interactions with other stakeholders, the volume changed before and after the COVID-19 pandemic, and the reason behind it. On-site observations were performed at different types of premises along the border, including farms, livestock markets, border control facilities, and the pathway where the smuggled cattle entered, and validated findings of KIIs and FGDs. The data collection process is shown in Figure 2.

First, held FGD seminars in counties and townships, gathering veterinary officials from the prefecture (superior level of the county), county, and township (the level below county). The participants were purposively selected, based on discussion with provincial CADC officials, to ensure participants had significant experiences working with the livestock industry. Oral consent was obtained from each participant at the beginning of the workshop, and then recorded in a list, after a brief introduction of the study and explicit explanation of the participant's rights. The participants were asked to identify the stakeholders involved in cross-border movement, raising and trade, and domestic transportation of the cross-border cattle.

Subsequently, KIIs were conducted individually at the participants “workplace.” Key informants were nominated during the FGDs and then invited through phone calls to obtain their agreement on voluntary participation. Each stakeholder identified was included in the nominations. The semi-structured interview approach was employed using open-ended questions to collect precise information on the joint and pathway of the smuggling cattle, and related practice on animal health, the volume of the smuggling cattle before and after the COVID-19 pandemic, and so on. The questions were piloted with three participants and were adjusted to minimize inappropriate interpretation.

Finally, personnel from the local Center for Disease Control and Prevention and investigators visited farms, trading markets, the local border and other places for on-site investigation and observation. They also had discussions and exchanges with local farm owners, management personnel, and trading personnel of the trading market to obtain the necessary information and data. The observations at each location were recorded in notes, photos, and video clips and were used to validate and complement the information that interviewees provided.

### 2.4. Data Analysis

Qualitative data collected from FGDs and KIIs were transcribed, systematically coded, and analyzed using thematic analysis. Themes were developed through both deductive reasoning, guided by the original research objectives, and inductive exploration, allowing new patterns and categories to emerge directly from the data. To minimize potential recall bias in historical reporting, particularly regarding cattle movement volumes, responses from different stakeholder groups were triangulated. Information provided by livestock brokers, transporters, veterinary personnel, and local officials was compared to identify inconsistencies. When significant discrepancies were detected, follow-up phone calls were conducted with respondents to clarify and verify the data. Quantitative data on cattle movement volumes were organized and analyzed using Microsoft Excel. Data from on-site observations were used for triangulation, ensuring consistency and reliability across data sources. Observational data were summarized and cross-referenced with reported stakeholder practices to validate findings.

## 3. Results

FGD summarizes the overall description of cross-border transportation in the study areas. A total of 25 veterinary officials participated in four FGDs (*n* = 7, 5, 6, and 7), and 54 stakeholders from different departments were interviewed individually, as shown in Table 1. On-site observations were conducted in seven farms, two livestock markets, and one border area, as shown in Table 2.

### 3.1. The Overall Information and Volume Changes of Cross-Border Movement of Cattle

#### 3.1.1. Before the COVID-19 Pandemic

① *Mengla County:* It covers a 740.8 km-long border, with six official and seven to eight unofficial animal cross-border routes. These routes are mainly distributed in Mengban Town and Mengman Town. Due to the lack of natural barriers and strict supervision, before the COVID-19 pandemic, residents in border areas could easily transport cattle into China. According to the memory of related stakeholders, approximately 200,000 head of cattle were illegally transported into Mengla County annually. ② *Menglong Town:* The majority of cross-border cattle into Jinghong City were through Menglong Town, which possesses a 78.39 km border devoid of natural barriers like rivers. Consequently, cattle can be easily transported into China via roadways, with each vehicle typically carrying six to eight cattle. Compared to Mengla County, the quantity and frequency of smuggled cattle in Menglong town are significantly higher. According to local cattle traders, the annual number of cattle smuggling in Menglong town was conservatively estimated at 300,000–600,000. ③ *Menghai County:* It spans a border of 146.56 km and lacks border physical barriers, allowing individuals on both sides of the Myanmar border to freely passage. Before COVID-19 pandemic, about 100,000 cattle were smuggled into Menghai County every year. ④ *Lancang County:* It has a border of 80.563 km, which provides convenience for cross-border smuggling of cattle through land transportation or artificial eviction. In addition, Lancang County has three large livestock markets, which promote livestock trade. Before COVID-19 pandemic, Lancang County smuggled at least 300,000 cows every year. ⑤ *Menglian County:* The border of Menglian County is 133.4 km long, with approximately 20 to 30 smuggling routes, allowing cattle to be driven to the area without restrictions. According to local cattle traders, about 350,000 to 600,000 cattle enter into Menglian County from Myanmar every year. The data is shown in Table 3.

#### 3.1.2. Post the COVID-19 Pandemic

During March and April 2020, in an effort to halt the cross-border transmission of COVID-19, the border areas of these counties established large-scale physical barriers, including barbed wire fencing (Figure S1). This significantly impeded previously accessible smuggling routes. Additionally, numerous surveillance cameras were deployed to monitor illegal activities, resulting in a marked decline in smuggling activities. Table 3 presents the variations in annual cattle smuggling volume within the investigated area, comparing pre- and post-COVID-19 pandemic conditions. The data obtained indicate that the COVID-19 pandemic and border control infrastructures have exerted a significant influence on cattle smuggling activities. These measures have effectively reduced the scale of cross-border animal movements, particularly smuggling activities. Consequently, there has been a notable decline in the number of smuggling incidents following the COVID-19 pandemic.

### 3.2. Paths of Cross-Border Animal Movement

Based on the information gathered from the surveyed counties, three primary routes for cross-border animal movement have been identified, each associated with a distinct main transit point (Table 4). The majority of the source countries for smuggled cattle are located in Southeast Asia and South Asia, with transportation methods encompassing both shipping and land transport.

#### 3.2.1. Path 1: Mainly Passing Through Mae Sot, Chiang Seng Port, and Sore Port

**Figure 3:**
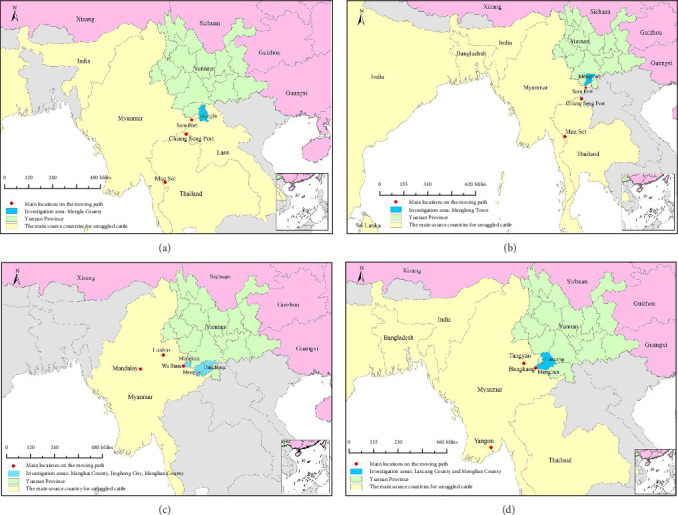
The movement path maps of smuggled cattle in research areas. (A) Cattle from India and Myanmar first arrive at Mae Sot and Chiang Seng Port in Thailand, along with Sore Port in Myanmar, before proceeding onward to Laos and Mongla County. (B) Cattle from countries such as India and Bangladesh, respectively, arrive at Mae Sot, Chiang Seng Port, and Sore port, subsequently arriving in Menglong Town. (C) Smuggled cattle traverse through Mandalay, Lashio, and Wa State, ultimately reaching Menghai County. (D) Cattle from other countries arrive in Yangon, Tangyan, and Bangkang in Myanmar, before proceeding to Lancang and Menglian Counties.

The smuggling routes of smuggled cattle in Mengla County and Menglong Town mainly pass through Mae Sot, Chiang Seng Port, and Sore Port; however, there are two counties where the cattle entered into China. ① *Entering Mengla County through Laos* (Figure 3A): Mengla County is close to Laos, but due to the relatively scarce number of cattle in Laos, the smuggled cattle in Mengla County are mainly imported from other countries through Laos. The specific route is that initially, cattle from other countries such as India and Myanmar were mainly transported to Thailand by water transportation. After centralized fattening in Mae Sot, Chiang Mai, Thailand, they were transported by water transportation to Chiang Seng Port in Chiang Rai and then shipped by boat along the Mekong River from Chiang Seng Port to Sore Port in Myanmar. From there, they were loaded onto trucks and transported to Laos. Finally, they are either transported by trucks or artificial eviction across the China–Laos border and eventually reach Mengla County. ② *Entering Menglong Town through Myanmar* (Figure 3B): Cattle from other countries such as India, Bangladesh, and Sri Lanka were initially brought to Thailand and then transported from Thailand to Myanmar before entering Menglong town. The specific route is as follows: first, cattle from other countries are mainly transported by water to the concentration of Mae Sot in Thailand, and then transported by water to the Chiang Seng Port in Thailand. From the Chiang Seng Port, they are transported upstream along the Mekong River to the port of Sore Port in Myanmar, and finally, these cattle enter Menglong Town. By boat in Thailand, by land in Myanmar, and then transported by road or directly artificial eviction after reaching the Yunnan border. It is worth noting that Sore Port is only 30 km away from the border of Menglong Town.

Although cattle from Mengla County and Menglong Town enter through Laos or Myanmar, respectively, the cross-border transportation routes are highly consistent. Cattle from other countries were initially transported to Thailand, which serves as a transit hub. After gathering in Mae Sot, Thailand, they were transported to the border port of Chiang Seng Port and then entered Myanmar. After arriving at the Sore Port in Myanmar, the route diverged. The cattle transported to Mengla County are transported to Laos and enter through the China–Laos border, as Laos is close to Mengla County. The cattle transported to Menglong Town directly enter China from Myanmar's Sore Port, as Sore Port is very close to Menglong Town.

#### 3.2.2. Path 2: Mainly Passing Through Mandalay, Lashio, and Wa State

The smuggling routes of smuggled cattle in Menghai County, a small part of Jinghong City, and a small part of Menglian County (Figure 3C) mainly pass through Mandalay, Lashio, and Wa State. The specific route of movement is for merchants to collect cattle in Mandalay and Lashio, Myanmar. These cattle are then transported by truck to the Wa State in Myanmar and then transported by land or directly by artificial eviction to the China–Myanmar border. Finally, they enter through Menghai County. This route, which passes through Mandalay, Lashio, Wa State, and the China–Myanmar border, is also one of the smuggling routes for transporting smuggled cattle to Jinghong City and Menglian County.

#### 3.2.3. Path 3: Mainly Passing Through Yangon, Tangyan, and Bangkang

The primary smuggling routes for cattle in Lancang and Menglian counties traverse through Yangon, Tangyan, and Bangkang, as illustrated in Figure 3D. The patterns of cattle smuggling in both counties exhibit consistency. Specifically, livestock originating from India, Bangladesh, Indonesia, and Thailand are initially conveyed via land or water transportation to Yangon. From Yangon, the cattle continue their journey overland to Tangyan. Subsequently, they are transported by vehicular means to Bangkang, and ultimately, into Lancang or Menglian counties, either through vehicular transportation or by manual eviction.

### 3.3. The Movement of Smuggled Cattle in China

**Figure 4:**
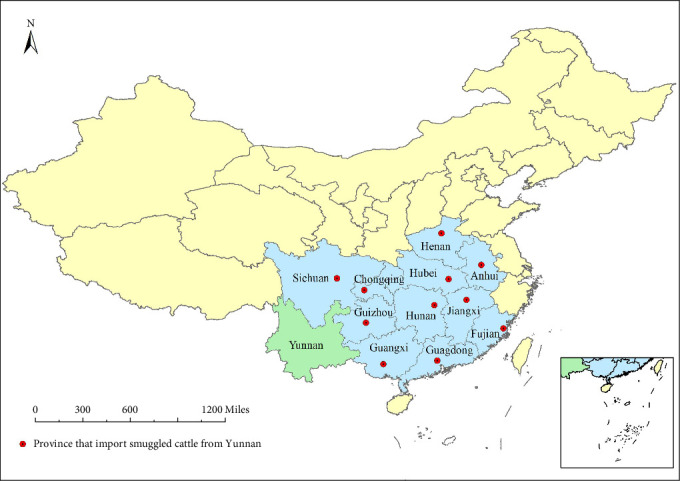
Major areas for the transportation and sale of smuggled cattle (represented by blue and red dots). A proportion of smuggled cattle in Yunnan Province circulate within the province, while others are sold throughout the country, especially in the southern regions.

The smuggled cattle not only circulate within Yunnan Province but are also traded to other provinces in China. According to local cattle traders, these animals are primarily sold to southern areas, including Guangdong, Guizhou, Hunan, Henan, Hubei, Sichuan, Chongqing, Anhui, Jiangxi, Fujian, and Guangxi (Figure 4). Buffaloes are predominantly sold to Anhui, Henan, Jiangxi, Guangdong, Sichuan, Chongqing, and Guangxi, while yellow cattle are mainly sold to Guangdong and Fujian. In contrast, other cattle breeds do not exhibit significant geospatial distribution characteristics.

### 3.4. Key Stakeholders, Premises, and Practices Contributing to Disease Transmission Risk

#### 3.4.1. Brokers

*(1) Cross-Border Brokers*: The transportation of smuggled cattle abroad primarily relies on cross-border brokers utilizing both water and land transportation. The hygienic conditions of ships and vehicles can significantly impact the animals' health. The absence of adequate cleaning and disinfection facilities on ships and vehicles fosters an environment that facilitates the spread of animal diseases. Moreover, the cattle originate from various countries, and the mixing of cattle from different origins during transportation further increases the risk of disease transmission. In addition, the tropical and subtropical climates and abundant forests in Southeast and South Asia are conducive to the survival and reproduction of pathogens and vectors, such as mosquitoes and biting midges, thereby increasing the risk of naturally occurring animal diseases and zoonotic diseases transmitted through vectors. Furthermore, a proportion of cattle were transported to the border areas by artificial eviction, which allows the cattle to have long-term contact and potentially contact with wild animals.

*(2) Domestic Venders*: The domestic vendors typically procured cattle from cross-border brokers and subsequently sold them at livestock markets. These vendors frequently travel between markets with differing hygiene standards, thereby elevating the risk of disease transmission via human and animal mobility. Furthermore, the sanitary conditions of the transportation vehicles are substandard, facilitating the survival and dissemination of pathogens. Moreover, domestic venders frequently utilize small barns for the temporary storage of unsold cattle. These barns are often cramped and unsanitary, and the cattle may hail from various sources, all of which contribute to the spread of disease. In addition, some vendors play multiple roles in the cattle value chain, including rearing, trading, transporting, and slaughtering.

#### 3.4.2. Live Cattle Market in Border Areas

During the research period, visits were made to two large-scale live cattle trading markets in Yunnan's border areas, one in Menghai County and the other in Menglian County. Both markets employ a combination of sheds and open-air spaces, with some cattle housed in sheds and others in open areas. The risks for disease transmission in live cattle markets stem from several factors:

① The significant volume of people and vehicles constantly entering and exiting these markets poses a challenge in maintaining adequate hygiene standards; ② The markets sell a wide range of cattle from different origins and breeds, leading to mixing that elevates disease risks; ③ The frequent trading and movement of cattle, both arriving and departing, exacerbate the risk of disease transmission; ④ Cattle dung is abundant throughout the markets, highlighting the necessity for enhanced sanitation and cleaning facilities; ⑤ The absence of troughs in the market, with forage left on the ground and trampled by both humans and cattle, makes it difficult to ensure forage hygiene.

## 4. Discussion

Border regions play a central role in the epidemiology of TADs, often bearing the brunt of transboundary livestock diseases [29]. The uncontrolled movement of people and animals along borders has been documented as one of the major factors for the introduction and continued circulation of animal diseases [30, 31]. While trade benefits border communities, it also creates or facilitates the transborder spread of livestock diseases [32]. This produces a continual introduction or reintroduction of the disease from either side owing to uncontrolled human and animal migration [29, 31]. Information on animal movements and interactions is critical in developing prevention, control and emergency plans for zoonotic diseases at the national, district, and subdistrict levels [33]. In order to prevent and control TADs, we must gather related information about the livestock cross-border movement and related behavior related to pathogen transmission. In this study, we investigated the activities of cattle smuggling along the border areas of Yunnan, China, with Myanmar and Laos. The investigation reveals that prior to COVID-19, millions of cattle were transported from Yunnan's border area into China. However, following the onset of COVID-19 pandemic and the establishment of physical barriers, the number of smuggled cattle drastically decreased. Most of the smuggled cattle originate from countries such as India, Bangladesh, and Myanmar. These cattle are typically brought to Thailand for short-term fattening before being transported into China via waterways and land routes through the borders with Laos and Myanmar. The smuggling operations follow relatively fixed transportation routes, and the majority of them are sold in southern provinces of China. Notably, numerous risk factors exist for the transmission of animal diseases throughout the entire value chain of cross-border cattle smuggling.

The demand fore beef and the price gap between China and adjacent Southeast Asian countries forced the cross-border trade and movement of livestock. The cattle breeding industry in Southeast or South Asia is relatively prosperous; however, the demand for beef in these countries is relatively lower. Therefore, the cattle price in these countries is generally lower than that in China. There was generally no natural barrier or sufficient border control infrastructure between counties in Yunnan; the number of cattle smuggled every year reached 1.75 million before COVID-19 [12]. According to villagers' recollections, cattle are carried by cars from the border areas almost every day, and foreign buffaloes and big-eared white cattle can be seen along the national highway. From March to April 2020, in order to prevent the spread of COVID-19, China set up border control infrastructures such as barbed wire, guard posts and cameras in the China-Myanmar and China-Laos border areas. Due to the erection of barbed wire and other devices in the border area, the number of imported cattle has sharply dropped. Besides, price is also an important factor. Since the beginning of March 2023, the domestic beef cattle prices continued to fall [34], and according to data from the National Bureau of Statistics in April 2024, the price of fattening cattle dropped to 30 yuan/kg, a new low in 4 years [35]. From 2022 to May 2024, the price of live cattle fell by 40%, and the price of beef fell by 30.1% [23]. However, the transportation and labor costs were increasing. Therefore, the narrowing of the price difference of cattle had also reduced the driving force of cross-boarding trade of cattle.

We have observed that Thailand has become a key transit and fattening hub for smuggled cattle. Additionally, the waterway transportation within Thailand is one of the most time-consuming parts of the cattle transport process. For effective prevention and control of TADs, it is crucial to closely monitor all stages of the process, including the origin of the cattle, the fattening process, transportation, and trade. Domestic movement, especially long-distance transportation, contributes to the spread of the TADs in domestic [36]. In this study, domestic trade and movement of these cattle were also described to analysis the possible exposure area. In China, most the of cattle are raised in the north, but beef resumption is more in the south, so nearly all of the abroad cattle were transported and sold in South China. Therefore, we must pay more attention to the monitoring and surveillance of TADs in these areas. The involvement of stakeholders in multiple, diverse roles, particularly in the context of cattle transportation, has been identified as a factor that exacerbates the risk of disease transmission [33]. In this study, it was observed that some vendors not only engage in the trade and transportation of cattle but also participate in livestock rearing and slaughtering. These vendors often possess breeding enclosures and transportation vehicles, and they commonly raise cattle for fattening purposes. It is estimated that approximately 10% of cattle weight is lost during transportation. To mitigate this loss and secure higher market prices, vendors typically raise the cattle for an additional 1–2 months. Consequently, greater attention must be given to the quarantine and vaccination of these animals, as well as to managing the multifaceted roles of stakeholders. Additionally, the movement of animals over long distances by farmers in search of pasture and water has been shown to increase interactions between animals, thereby facilitating the transmission of infectious diseases [37]. Therefore, the practice of cross-border brokers hiring farmers to drive cattle across borders further amplifies the risk of disease spread. In summary, the activities of vendors and brokers are critical factors that can aggravate the transmission of infectious diseases in livestock populations.

It has been indicated that livestock markets are one of the major factors contributing to the circulation and distribution of animal diseases, such as FMD, in sub-Saharan Africa [29]. According to our survey, there are many large and small livestock markets in the border area. In the border county, there is at least one major live cattle market. Inadequate cleaning and lack of disinfection represent significant risk behaviors for the spread of pathogens in these markets. Previous studies have also shown that joint herding for grazing and the exchange of bulls facilitate the rapid spread of diseases [37, 38]. In our research, we observed the practice of gathering cattle from different batches in a single vehicle and combining herds for grazing, which may further promote interherd contact and disease transmission.

The TADs are a major threat to livestock of any nation, as they have the potential to cause large-scale damage, staking the food security of the country, and can cripple the nation's economy significantly by direct loss in the form of disease morbidity and mortality in an affected population or indirectly due to required counter-epizootic measures, loss in trade, and probable zoonotic transmission [2]. Besides, the One Health approach to preventing the spread of zoonotic diseases, especially cross-border, is an emerging topic of interest to multiple sectors and agencies [33]. Therefore, to ensure the safety of livestock and public health, cross-border and cross-sector cooperation is essential for the prevention and control of TADs. The study indicated that a significant number of cattle entered China through the borders with Laos and Myanmar every year before COVID-19. Although the volume of cattle has sharply decreased since the pandemic, the risk of TADs should not be underestimated. We have observed that some quarantine facilities in the border areas are beginning to show signs of deterioration. According to local villagers, some of this damage was intentionally caused by individuals involved in cattle smuggling, while other damage resulted from natural factors. The price of cattle continues to fluctuate, and as long as there is market demand and a price differential, smuggling activities are unlikely to cease. Therefore, it remains crucial to closely monitor cross-border cattle smuggling activities.

This study has several limitations that may affect the interpretation of its findings. First, not all border areas in Yunnan Province were covered by the study, which may limit the generalizability of the results; however, selected study areas have significant representatives according to previous investigations conducted by local veterinary authorities. Second, the accuracy of information obtained during the investigation may be affected by potential recall biases from respondents, particularly movement volumes. To mitigate this, a triangulation strategy was applied by gathering information from a diverse range of stakeholders and cross-validating the data across sources.

Building on the results of this study, we propose several key areas for further research to enhance the prevention and control of TADs. First, it is crucial to develop longitude surveillance system capable of monitoring cross-border animal movements over extended time periods. Second, targeted strategies to improve animal management and enhance biosecurity measures should be explored, particularly in relation to the high-risk practices identified. Additionally, the application of spatial epidemiological methods can support the identification of domestic and transboundary risk zones, thereby facilitating effective zoning management. Further research in these areas will be critical for mitigating the risks of TADs in border areas.

In conclusion, the cross-border movement of cattle along the border areas of Yunnan, China, with Myanmar and Laos poses a significant risk to the control of TADs. Regular risk assessments and the implementation of continuously targeted measures are essential to address this issue effectively.

## Figures and Tables

**Table 1:** Socialdemographic information of the participants of focus group discussions (*n* = 25) and key information interviews (*n* = 54) conducted in Yunnan in 2023.

Type of institutions (number of institutions)	Role of participants in the institute	Number of participants	Gender of participants (male = M and female = F)
Focus group discussions (FGDs) *(n* = 25)			
County level veterinary institutions (2)	Veterinary officers	12	10M, 2F
Township level veterinary institutions (2)	Veterinary officers	13	11M, 2F
Key information interviews(KII) (*n* = 54)			
Farms (7)	Farmers	7	23M, 4F
	Other related staffs	20	
Brokers	Brokers	5	5M
Live cattle trading markets (2)	Managers	4	4M
	Trading person	9	7M, 2F
Border institution (1)	Border police station person	3	3M
State/municipal veterinary institutions (2)	Veterinary officers	5	2 M, 3F
Provincial veterinary institution (1)	Veterinary officer	1	1 M

**Table 2:** Survey locations of research areas.

Type of institutions (number of institutions)	Investigation location
Farms (7)	• Farm A, Mengla County
	• Farm B, Farm C, Menglong Town
	• Farm D, Farm E, Lancang County
	• Farm F, Farm G, Menglian County

Live cattle trading markets (2)	• Market A, Menghai County
	• Market B, Menglian County

Border institution (1)	• Border Police Station A, Mengla County

**Table 3:** The change of annual cattle smuggling volume in the surveyed area before and after COVID-19.

Survey area	Pre-COVID-19 (head/year)	Post-COVID-19 (head/years)	Magnitude of change
Mengla County	200,000	—^a^	—
Menglong Town	300,000–600,000	6000–12,000	−98%
Menghai County	100,000	0	−100%
Lancang County	300,000	—	−80% to −90%^b^
Menglian County	350,000–600,000	—	−94 to −96%^b^

^a^Not gained.

^b^Estimated by the changes in livestock trading volume. The number of smuggled cattle has significantly decreased after COVID-19.

**Table 4:** Basic information on the smuggling routes of smuggled cattle in the investigation areas.

Investigation area (border)	The main source country for smuggled cattle	Transport mode	Main location along the route
Mengla County (route 1)	Myanmar, India, Thailand, Laos	Water transportation, land transportation, manual eviction	Mae Sot, Chiang Seng Port, Sore Port, border between China and Laos
Menglong Town (route 1)	Myanmar, India, Thailand, Bangladesh, Sri Lanka	Water transportation, land transportation, artificial eviction	Mae Sot, Chiang Seng Port, Sore Port, the border between China and Myanmar
Menghai County (route 2)	Myanmar	Land transportation, artificial eviction	Mandalay, Lashio, Wa State, the border between China and Myanmar
Lancang County, Menglian County (route 3)	Myanmar, Thailand, India, Bangladesh, Indonesia	Water transportation, land transportation, artificial eviction	Yangon, Tangyan, Bangkang, the border between China and Myanmar

*Note:* The smuggled cattle in the investigation areas primarily originate from Southeast Asian and South Asian countries, entering through various means including water transportation, land transportation, and artificial eviction.

## Data Availability

The data will be made available upon request.

## Supporting Information

Supporting Information Figure S1: Isolation Facilities at the China-Laos Border. Figure S1 shows the border physical barriers between China and Laos. In order to prevent the spread of the disease after the epidemic, the government has set up a large-scale of barbed wire barriers, large iron gates, and fences at the border between Mengla County and Laos. According to local villagers and staff, similar border control infrastructures have been set up in all border counties in Yunnan Province after the epidemic, which has had a significant impact on smuggling activities.
