# Supplementary figures and images for: Analysis of Cross-Border Movement of Cattle in Yunnan Province, China: Insights Into Transboundary Animal Diseases Control
Source: Transbound Emerg Dis. 2025 Sep 11;2025:5561414. doi: 10.1155/tbed/5561414 (PMC12446582; doi:10.1155/tbed/5561414)

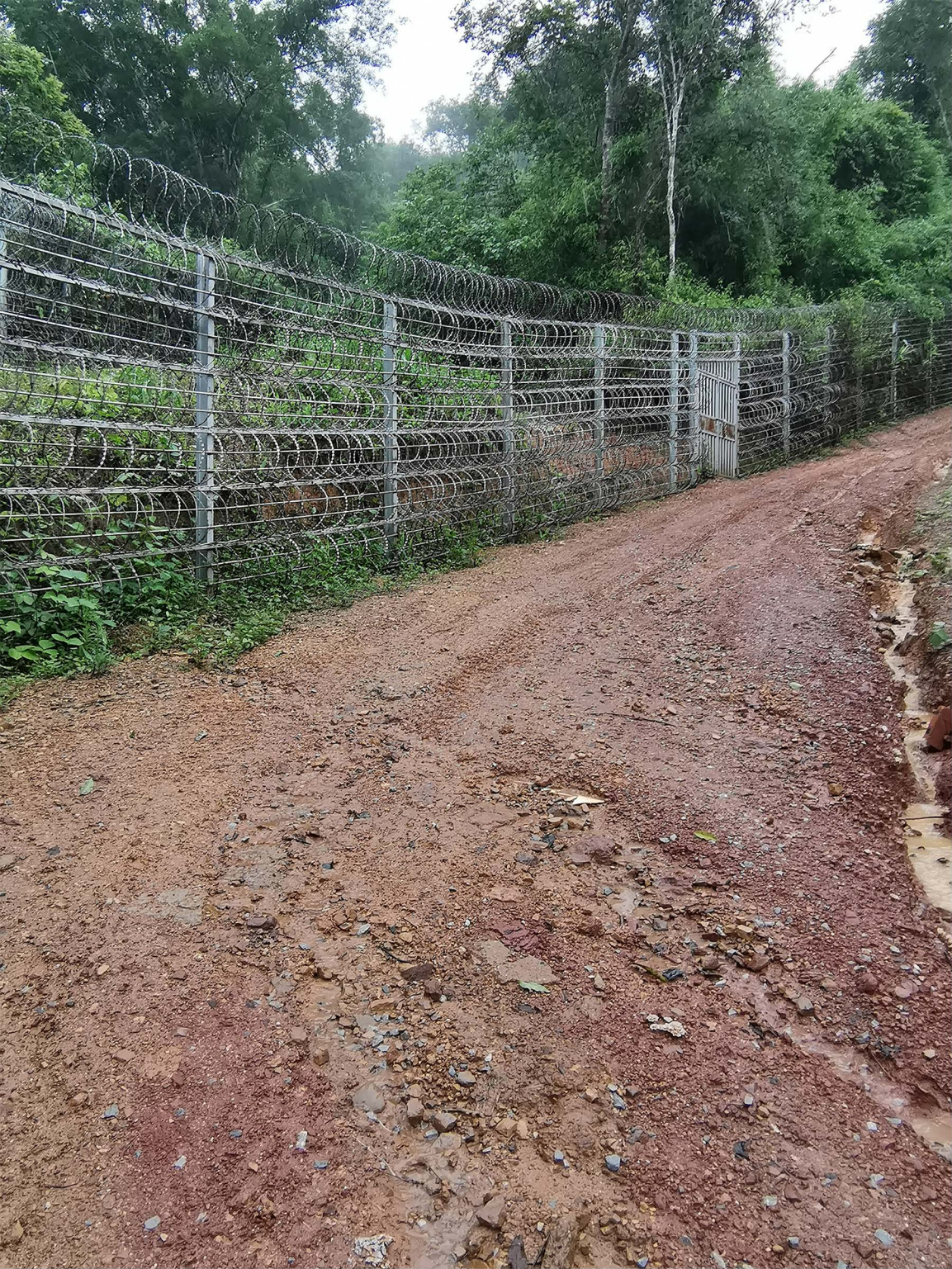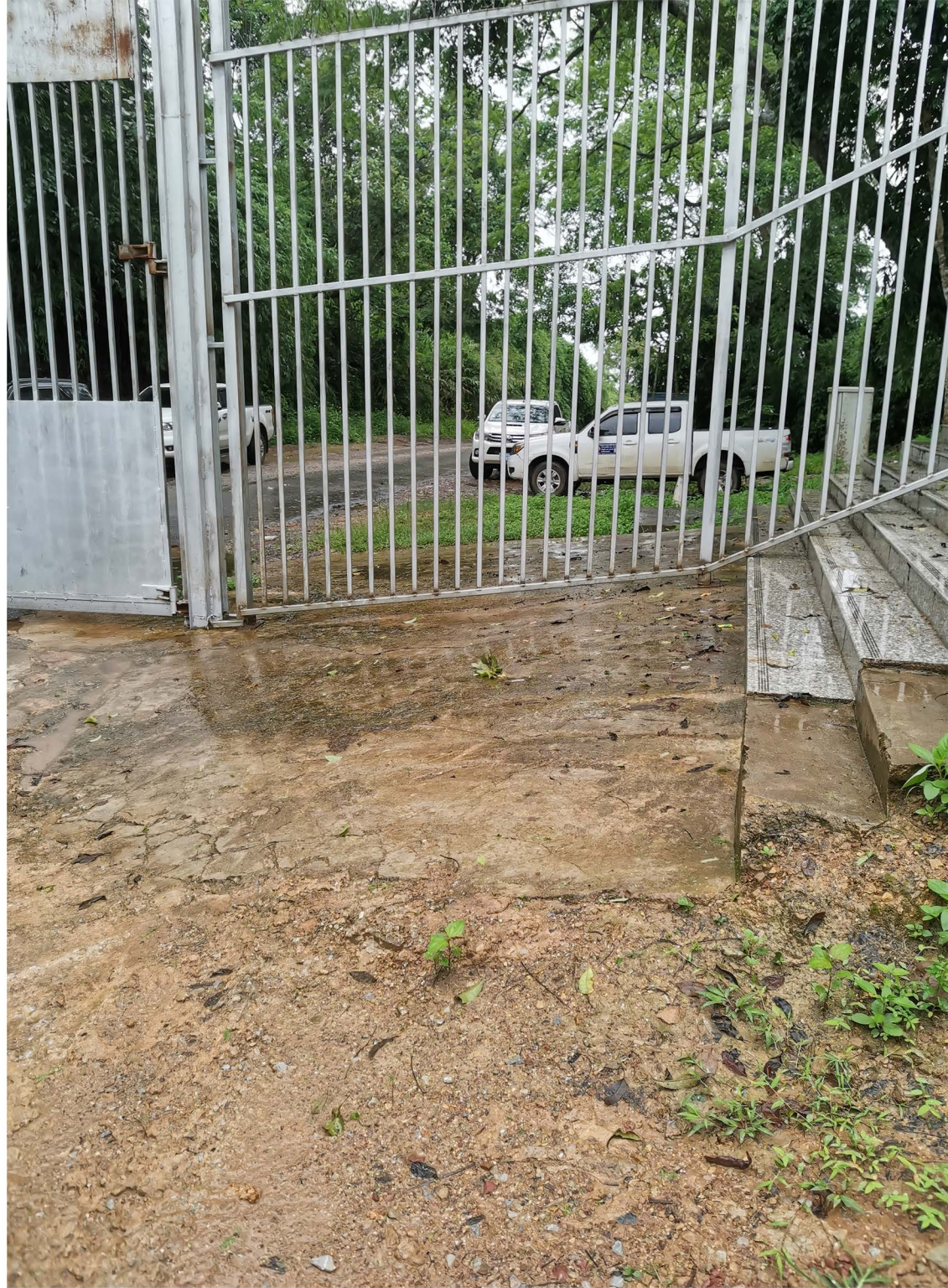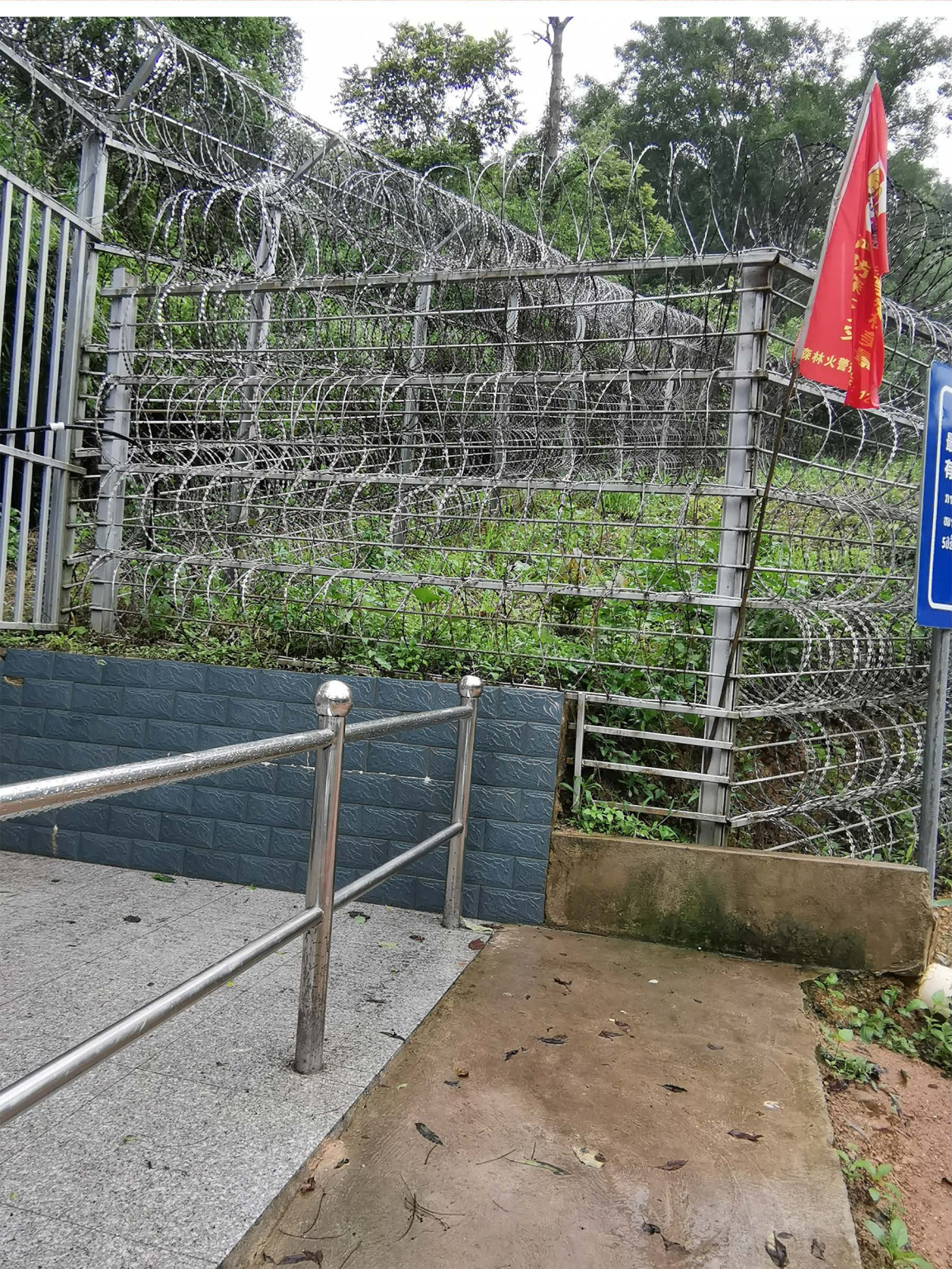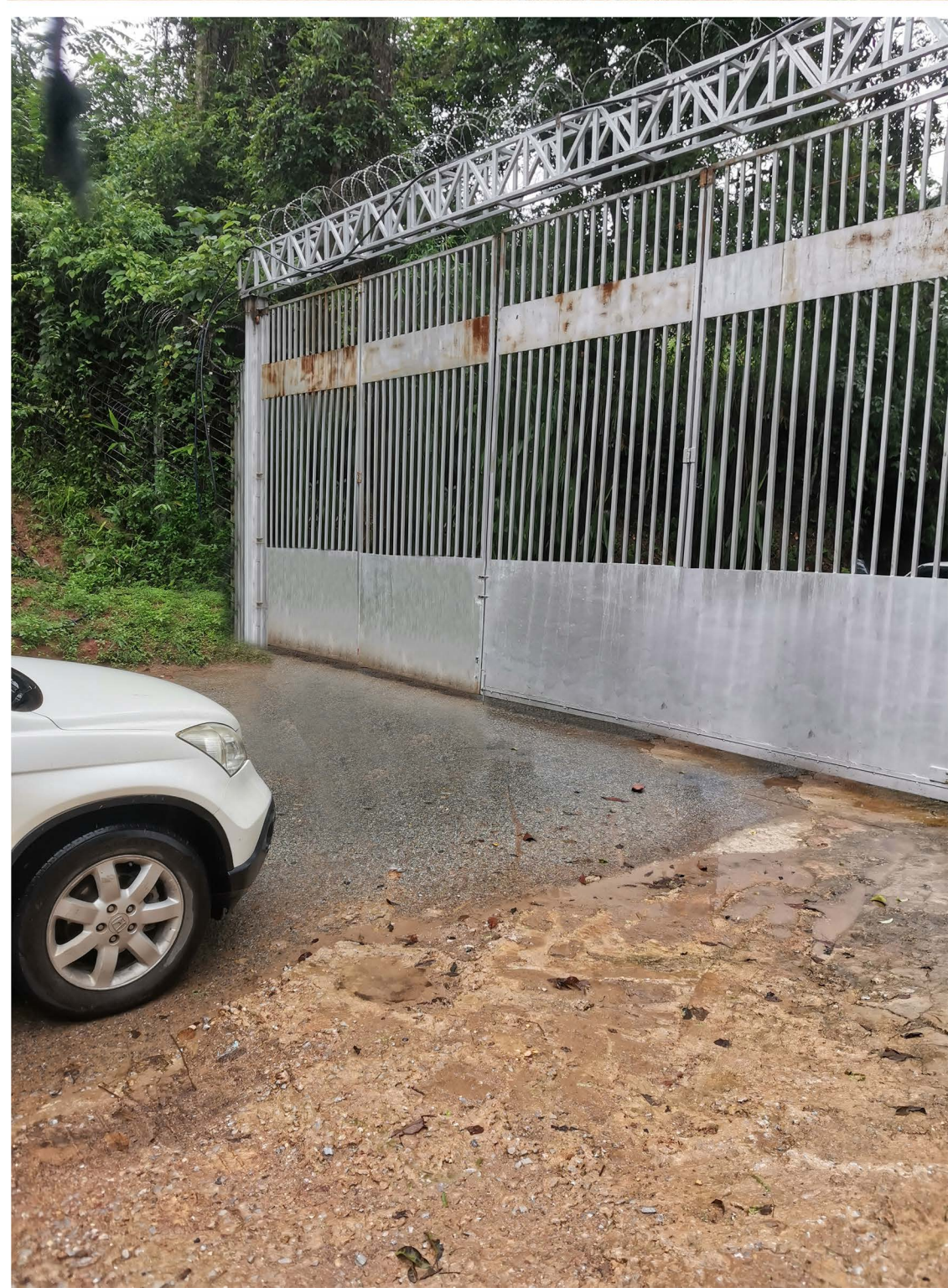

Supplement: Supporting Information — Figure S1: Isolation Facilities at the China-Laos Border. Figure S1 shows the border physical barriers between China and Laos. In order to prevent the spread of the disease after the epidemic, the government has set up a large-scale of barbed wire barriers, large iron gates, and fences at the border between Mengla County and Laos. According to local villagers and staff, similar border control infrastructures have been set up in all border counties in Yunnan Province after the epidemic, which has had a significant impact on smuggling activities. [file 5561414.f1.pdf]
